# Dissecting the inflammatory tumor microenvironment of esophageal adenocarcinoma: mast cells and natural killer cells are favorable prognostic factors and associated with less extensive disease

**DOI:** 10.1007/s00432-023-04650-0

**Published:** 2023-02-24

**Authors:** Alyne Condurú dos Santos Cunha, Adrian Georg Simon, Thomas Zander, Reinhard Buettner, Christiane Josephine Bruns, Wolfgang Schroeder, Florian Gebauer, Alexander Quaas

**Affiliations:** 1 Institute of Pathology, University Hospital Cologne, Medical Faculty, University of Cologne, Kerpener Str. 62, DE-50937 Cologne, Germany; 2 Department of General, Visceral and Cancer Surgery, University Hospital Cologne, Medical Faculty, University of Cologne, Cologne, Germany; 3 Department of Internal Medicine I, University Hospital Cologne, Medical Faculty, University of Cologne, Cologne, Germany

**Keywords:** Esophageal cancer, Esophageal adenocarcinoma, Mast cells, Tumor microenvironment, Natural killer cells, Tumor immune environment

## Abstract

**Purpose:**

Esophageal adenocarcinoma (EAC) remains a challenging and lethal cancer entity. A promising target for new therapeutic approaches, as demonstrated by the success of immune checkpoint inhibitors, are tumor-associated immune cells and the tumor microenvironment (TME). However, the understanding of the TME in esophageal cancer remains limited and requires further investigation.

**Methods:**

Over 900 EAC samples were included, including patients treated with primary surgery and neoadjuvant (radio-)chemotherapy. The immune cell infiltrates of mast cells (MC), natural killer cells (NK cells), plasma cells (PC), and eosinophilic cells (EC) were assessed semi-quantitatively and correlated with histopathological parameters and overall survival (OS).

**Results:**

A high presence of all four immune cell types significantly correlated with a less extensive tumor stage and a lower frequency of lymph node metastasis, and, in case of NK cells, with less distant metastasis. The presence of MC and NK cells was favorably associated with a prolonged OS in the total cohort (MC: *p* < 0.001; NK cells: *p* = 0.004) and patients without neoadjuvant treatment (MC: *p* < 0.001; NK cells: *p* = 0.01). NK cells were a favorable prognostic factor in the total cohort (*p* = 0.007) and in the treatment-naïve subgroup (*p* = 0.04). Additionally, MC were a favorable prognostic factor in patients with lymph node metastasis (*p* = 0.009).

**Conclusion:**

Our results indicate a complex and important role of mast cells, NK cells, and the other assessed immune cells in the tumor microenvironment of EAC. Therefore, they are one further step to a better understanding of the immune cell environment and the potential therapeutic implications in this cancer entity.

## Introduction

Despite recent advances in surgery, endoscopy and immunotherapy, esophageal cancer remains a challenging and deadly disease. Esophageal squamous cell carcinoma (ESCC), the most common subtype worldwide, accounts for over 85% of all cases globally, especially in central- and south eastern Asia (Thrift 2021). However, likely due to the varying geographical distribution of risk factors, the incidence of esophageal adenocarcinoma (EAC), the second most common subtype, is rapidly rising in Europe and Northern America, partly even surpassing ESCC (Arnold et al. 2017). Since both esophageal cancer subtypes usually remain symptomless for a long time, patients often present in an advanced stage and have a dismal prognosis: Even if treated with curative intent, including neoadjuvant (radio-)chemotherapy followed by extensive surgery, only 43% of the patients survive 5 years; inoperable patients, however, with only palliative treatment options left, usually survive few months (Rubenstein and Shaheen 2015; Dijksterhuis et al. 2020, Eyck et al. 2022).

Thus, there is an urgent need for a better understanding of these cancer entities as well as for additional therapeutic approaches. One effective target, as demonstrated by the impressive success of immune checkpoint inhibitors in past years, is the tumor immune cell environment. As one example among many, the functional as well as the prognostic significance of T-lymphocytes in microsatellite-unstable colon carcinomas has been demonstrated and highlighted (Kakar et al. 2004; Galon et al. 2006) and led to a practical approach with immune checkpoint inhibitors, showing first promising results in treatment for this cancer (Andre et al. 2021).

Compared to lymphocytes, the research data on presence, function, and relevance of other inflammatory cells in esophageal cancer are limited, especially in EAC: Our research group has recently described a partly sex-dependent prognostic significance of neutrophil granulocytes in carcinoma entities of the upper gastrointestinal tract (Abdel-Latif et al. 2004; Hu et al. 2015; Clausen et al. 2020; Quaas et al. 2021). In this study, we continue our analysis in EAC assessing mast cells (MC), natural killer cells (NK cells), plasma cells (PC) and eosinophilic cells (EC), thus further closing one more gap to a better understanding of the complex inflammatory microenvironment of these cancer entities.

## Materials and methods

### Patient cohort and histopathological data

In this study, 953 patients were included who were treated between 1996 and 2020 at the Department of General, Visceral and Cancer Surgery at the University Hospital of Cologne. All patients were treated for adenocarcinomas of the esophagus. Written consent was obtained by all patients, the ethics committee of the University Hospital of Cologne approved the project (ethics committee number: 20-1393).

The patient cohort consisted of 836 male patients (88.72%) and 117 female patients (12.27%) with a median age at surgery of 63 years (27–91 years) (Table 1). The patients received either primary surgery (*n* = 320, 33.58%) or, in clinically advanced stage (cT3, cNx, M0), neoadjuvant treatment followed by surgery (*n* = 633, 66.42%) (Table 1): In 322 cases (33.79%), patients were treated according to the CROSS standard (chemoradiation, followed by surgery), in 99 cases (10.39%) a FLOT chemotherapy (fluororuracil, leucovorin, oxaliplatin, docetaxel) was used as neoadjuvant treatment. In 212 cases (22.25%), the neoadjuvant treatment varied from CROSS or FLOT scheme and was not further specified. The standard surgical procedure consisted of right transthoracic esophagectomy and a two-field lymphadenectomy of mediastinal and abdominal lymph nodes. A surgical reconstruction of the intestinal passage was performed by a high intrathoracic esophagogastrostomy as described previously (Holscher et al. 2007).

**Table 1:** Histopathological parameters of the patient cohort (*n* = 953)

		Primary surgery	Neoadjuvant treatment		Total*
			CROSS	FLOT	
		*n* (%)	*n* (%)	*n* (%)	*n* (%)
Sex					
Male		279 (87.19)	280 (87.58)	89 (89.90)	836 (88.72)
Female		41 (12.81)	40 (12.42)	10 (10.10)	117 (12.27)
Age at surgery (years)					
Median (range)		67 (30–91)	60 (27–84)	61 (34–86)	63 (27–91)
pT/ypT					
pT1/ypT1		113 (35.31)	33 (10.25)	10 (10.10)	180 (18.89)
pT2/ypT2		59 (18.44)	62 (19.25)	15 (15.15)	177 (18.57)
pT3/ypT3		140 (43.75)	223 (69.25)	68 (68.69)	565 (59.29)
pT4/ypT4		8 (2.50)	4 (1.25)	6 (6.06)	31 (3.25)
pN/ypN					
pNx/ypNx		1 (0.3)	0 (0.0)	0 (0.0)	1 (0.1)
pN0/ypN0		141 (44.06)	136 (42.23)	33 (33.33)	391 (41.02)
pN + /ypN +		178 (55.63)	186 (57.76)	66 (66.67)	561 (58.87)
L					
Lx		80 (25.00)	43 (13.35)	0 (0.0)	163 (17.10)
L0		122 (38.13)	178 (55.28)	46 (46.46)	435 (45.65)
L1		118 (36.88)	101 (31.37)	53 (53.54)	355 (37.25))
V					
Vx		79 (24.69)	41 (12.73)	0 (0.0)	158 (16.58)
V0		207 (64.69)	259 (80.43)	88 (88.89)	704 (73.87)
V1		34 (10.63)	22 (6.83)	11 (11.11)	91 (9.55)
Pn					
Pnx		77 (24.06)	40 (12.24)	0 (0.0)	153 (16.05)
Pn0		188 (58.75)	230 (71.43))	65 (65.66)	615 (64.53)
Pn1		55 (17.19)	52 (16.15)	34 (34.34)	185 (19.41)
M					
Mx/M0		291 (90.94)	317 (98.45)	97 (97.98)	904 (94.86)
M1		29 (9.06)	5 (1.55)	2 (2.02)	49 (3.56)
Stage (UICC)					
I		88 (27.50)	22 (6.83)	7 (7.07)	133 (13.93)
II		44 (13.75)	38 (11.80)	11 (11.11)	123 (12.88)
III		116 (36.25)	161 (50.0)	41 (41.41)	427 (44.71)
IV		72 (22.50)	101 (31.37)	40 (40.40)	272 (28.48)
AJCC grade**					
NA		6 (1.80)	–	–	
Grade 1		3 (0.94)	–	–	
Grade 2		162 (50.63)	–	–	
Grade 3		146 (45.63)	–	–	
Grade 4		3 (0.94)	–	–	

Patient cohort: *n* = 953

*Patients pre-treated with other therapeutic approaches than CROSS or FLOT (*n* = 212) are not displayed in this overview, but are taken into the total column

*CROSS* chemoradiotherapy for esophageal cancer followed by surgery study; *FLOT* fluorouracil, leucorovin, oxaliplatin, docetaxel, followed by surgery; TNM classification was performed following UICC 2020, 8th edition criteria

**AJCC grading was not performed for tumors treated with neoadjuvant therapy. *NA* not assessed

In the first 2 years after surgery, a clinical followed-up was performed every 3 months, followed by annual examinations afterward. The clinical follow-up included a detailed medical history since surgery, physical examination, an ultrasound scan of the abdomen, a chest X-ray and, if required, additional diagnostics.

### Generation of tissue microarrays

To assess as many patient samples as possible in a concise, yet comprehensive approach, tissue microarrays (TMAs) were generated using formalin-fixed, paraffin-embedded tumor tissue (Simon et al. 2004). In this study, two tissue cores of each tumor with a diameter of 1.2 mm were transferred from diagnostic paraffin blocks to the recipient paraffin block using a self-constructed semi-automated precision instrument. The tissue cores were carefully selected from representative areas of the tumor by an expert gastrointestinal pathologist (AQ) (see below). To later verify and assess the quality of the immunohistochemical staining, tonsil tissue was included as control tissue.

### Standard H&E and immunohistochemical staining of the tissue microarrays

To assess the inflammatory and immune cell infiltrate in esophageal adenocarcinoma tissue, in a first step, the TMAs were stained with hematoxylin–eosin following standard protocols in the routine laboratory at the Institute of Pathology, Cologne University Hospital. For plasma cells and mast cells, additional immunohistochemical staining was performed: Plasma cells were stained using an antibody against CD38 (CD38-290-L-CE, 1:800, Novocastra by Leica Biosystems, Wetzlar, Germany). Mast cells were stained for CD117 (CD117/c-kit, 1:50, Biocare Medical, Pacheco, USA). Natural killer cells were stained for CD56 (CD56 monoclonal antibody 123C3, 1:500, Thermo Fisher, Waltham, USA). The immunohistochemical staining was performed on a Bond staining platform (Leica, Wetzlar, Germany) following well-established routine protocols of the Institute of Pathology at Cologne University Hospital.

### Assessment of inflammation and immune cells

A trained medical observer and an experienced pathologist independently assessed the immune cell infiltrate in the esophageal adenocarcinoma specimens. The extent of the inflammatory cells was determined using a semi-quantitative approach as described before (Quaas et al. 2021). Shortly summarized, the immune cell infiltrate was assessed software assisted in the first 200 carcinoma samples: Tumors displaying null to three cells per high-powered field were classified as negative (0). The cell count above three cells then was dichotomized in a low-level infiltrate (4–10 cells/mm^2^, 1 +) and high-level infiltrate (> 10 cells/mm^2^ 2 +). This software-assisted approach was followed by a semi-quantitative evaluation performed by two expert pathologists and had a concordance of 95% with the software-assisted approach. After assessment of these three cores, an average value for the total tumor was generated. We further analyzed whole sections of 20 adenocarcinomas to assess the representativity of TMA cores. Five tumor samples were selected that had high or low levels of plasma cells and mast cells, respectively. These whole sections were stained with CD117 (mast cells) and CD38 (plasma cells) and the infiltrate was assessed by two trained observers regarding their immune cell amount, distribution, and similarity to the TMA excerpts. The whole sections were in concordance with the TMA cores, so overall we believe that the technique of TMA is able to provide adequate data.

### Statistical analysis

Interdependencies between the extent of the specific immune cell infiltrate, patient characteristics (age, sex), and histopathological tumor characteristics (TNM, UICC) were analyzed using Fisher’s exact test and Spearman correlation with Benjamini–Hochberg correction for multiple comparisons. The immune cell infiltrate was dichotomized into negative tumors and tumors with low level infiltrate (0 = negative, 1 +  = low level) versus tumors with high immune cell infiltrate (2 +  = high level). In case of NK cells and EC, which comprised a very low number of tumors with high infiltrate, the cohort was dichotomized in negative (0) and positive (1 + , 2 +) tumors. To assess the correlation between immune cell infiltrate in EAC and survival as well as the prognostic impact, the overall survival (OS) was evaluated from the date of surgery until death (of any cause). Kaplan–Meier curves were generated and a log-rank test or a Breslow test were performed, depending on the shape of the Kaplan–Meier curves and the assumptions of proportional hazards: Patient data with no events or lost clinical follow-up were censored at the last known date. Univariate analysis, followed by multivariate analyses were performed using a Cox regression model (Enter method): Beside the immune cell infiltrate, patient age, sex, histopathological factors affecting survival (pT, pN, L, V, Pn), AJCC grade, and the application of neoadjuvant therapy were included. A two-sided *p* value < 0.05 was considered to be statistically significant, a *p* value < 0.1 as a statistical trend. For all data preparation, statistical analysis and data visualization Python v.3.9 and PyCharm (Community Edition v.2022.2) were used, including openly available common packages for statistics and data visualization (numpy, pandas, scipy.stats, matplotlib, pingouin, lifelines).

## Results

### Basic characteristics and immune cell environment in the patient cohort

**Fig. 1:**
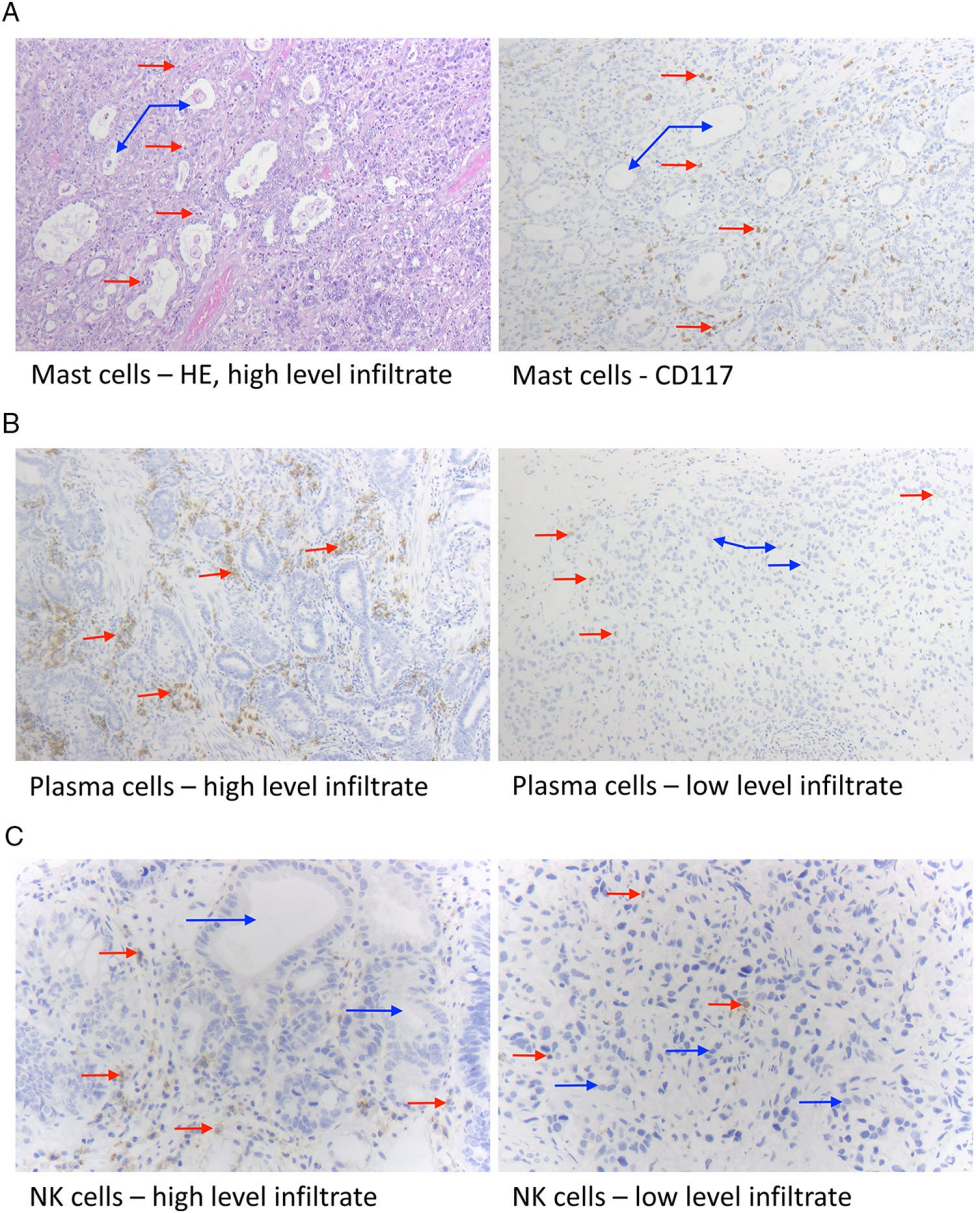
Histological and immunohistochemical staining results. Esophageal adenocarcinoma, as displayed here, consists of atypical, often bizarrely formed glands, infiltrating the wand layers of the esophagus (blue arrows). The cells display a vary in form and size and have pleomorphic nuclei. The red arrows show the specific cell type of the inflammatory tumor environment: **A** Mast cells, HE staining, and immunohistochemistry for CD117; 100 × magnification; **B** plasma cells (CD38 staining), 100X; **C** natural killer cells, immunohistochemistry for CD56, 200X

In this study, 921 out of the 953 included tumor samples (97.17%) were successfully evaluated for tumor-infiltrating mast cells (MC), 926 cases (97.07%) for natural killer cells (NK cells), 923 samples (96.85%) for plasma cells (PC), and 916 patients (96.12%) for eosinophils (EC) (Fig. 1, Table 2). Reasons for the non-informative cases were missing cores in the paraffin block or the absence of any cancer tissue in the TMA. Except for PC and EC, tumors with an extensive intra-tumoral infiltrate of one immune cell type typically displayed concurring high levels of the other assessed immune cells (Table 3). A significant difference in the immune cell extent of MC, NK cells, PC, and EC depending on age and sex was not observable (data not shown).

**Table 2:** Composition of the immune cell infiltrate in the EAC cohort

	MC	NK cells	PC	EC
	*n* (%)	*n* (%)	*n* (%)	*n* (%)
Cell infiltrate				
Negative (0)	274 (29.75)	539 (58.10)	445 (48.21)	761 (82.86)
Low (1)	470 (51.03)	373 (40.28)	353 (38.24)	156 (17.03)
High (2)	177 (19.2)	15 (1.62)	125 (13.54)	1 (0.11)
Negative (0)		539 (58.10) *		761 (82.86) *
Positive (1 + 2)		388 (41.90) *		157 (17.14) *
Total	922 (100)	926 (100)	923 (100)	916 (100)

*MC* mast cells, *NK cells* natural killer cells, *PC* plasma cells, *EC* eosinophils

*For NK cells and EC, only very few tumors displayed high levels (2+) of immune cell infiltrate. For further statistical analysis, these cohorts were dichotomized in negative (0) tumors versus positive tumors (1 +  = low, 2 +  = high level)

**Table 3:** Interrelationships between specific immune cell infiltrates in esophageal cancer

Cell type	MC	NK cells	PC	EC
MC				
*p* value	-	< 0.001	< 0.001	< 0.001
NK cells				
*p* value	< 0.001	-	0.008	0.01
PC				
*p* value	< 0.001	0.008	-	0.08
EC				
*p* value	< 0.001	0.01	0.08	-

*MC* mast cells, *NK cells* natural killer cells, *PC* plasma cells, *EC* eosinophilic cells; The distribution of low/high levels of MC and PC and positive/negative tumors in case of NK cells and EC was analyzed using Fisher’s exact test with Benjamini–Hochberg correction. A *p* value < 0.05 was considered statistically significant

### Extensive infiltrate of immune cells correlates with less extensive disease

The tumor samples where dichotomized, in case of MC and PC into a negative/low infiltrate- and high infiltrate-subgroup, in case of NK cells and EC into a negative and a positive subgroup (Table 3). Extensive infiltrates of MC, NK cells, PC, and EC were associated with mucosa- and submucosa-limited tumors (pT1); patients with immune cell-negative tumors or sparse cell infiltrate had significantly more often extensive, deeper infiltrating cancers (pT2, pT3, pT4) (MC: *p* < 0.001; NK cells: *p* = 0.003; PC: *p* < 0.001; EC: *p* < 0.002; Fisher’s exact test, Benjamini–Hochberg correction). Additionally, patients with higher levels of MC and presence of NK cells and EC less often had lymph node metastasis (pN0 vs pN1, pN2, pN3) (MC: *p* < 0.001; NK cells: *p* = 0.002; EC: *p* = 0.002). Negativity for NK cells was associated with higher frequency of lymph vessel invasion (L) (*p* = 0.008), occurrence of distant metastasis (*p* = 0.02), and a higher AJCC grade (*p* = 0.02). Patients with low levels of plasma cell infiltrate significantly more often displayed perineural invasion (*p* = 0.012).

In concordance with all these findings, high inflammatory infiltrate levels were significantly more frequent in patients with low UICC stage (MC: *p* < 0.001; NK cells: *p* = 0.003; PC: < 0.001; EC: *p* < 0.001).

In summary, patients with low levels or absence of all assessed immune cell types had deeper infiltrating, more extensive tumors, and in case of MC, NK cells, and EC, more often lymph node metastasis.

### High levels of immune cells are correlated with longer overall survival

**Fig. 2:**
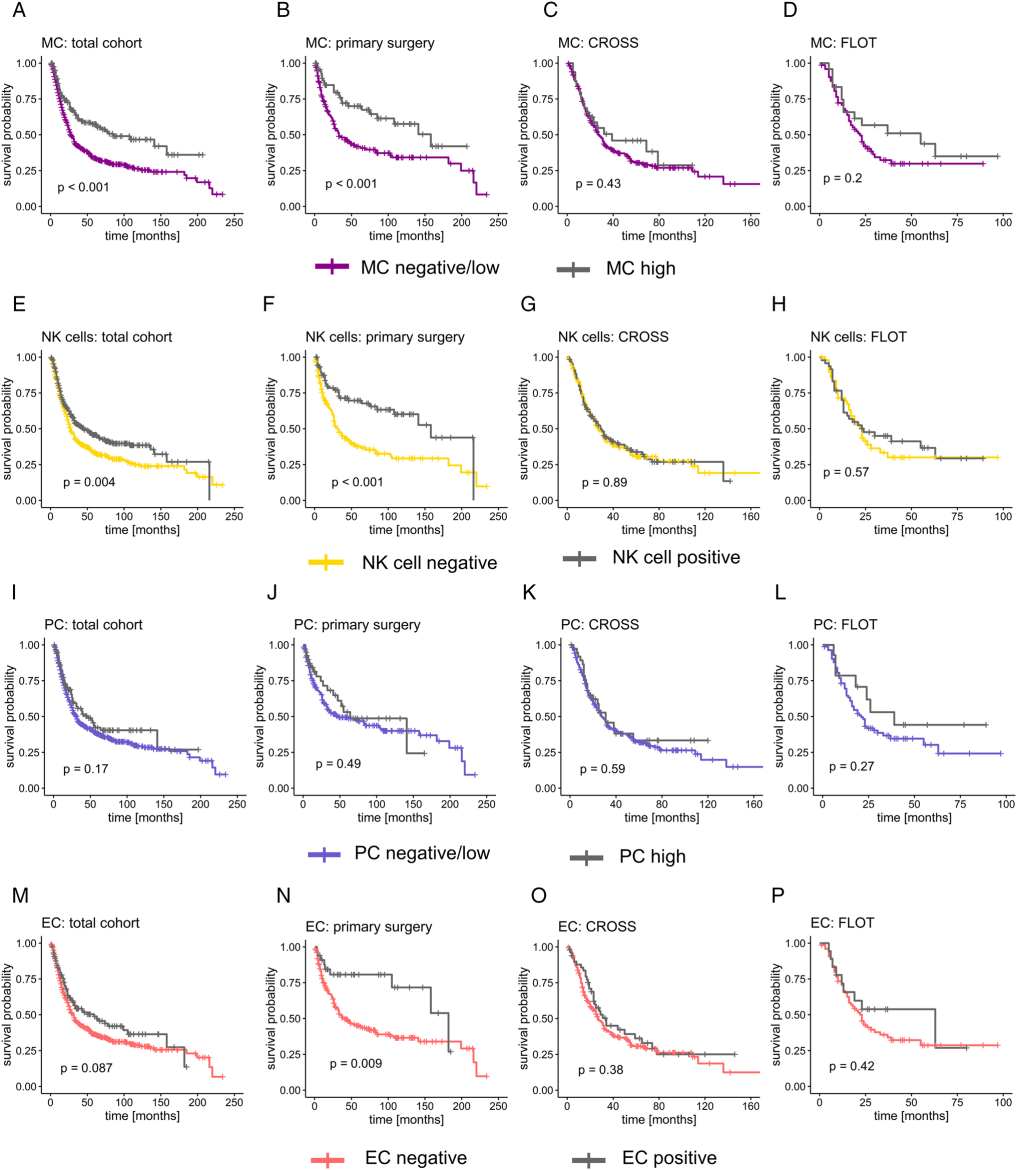
Correlation of immune cell infiltrate and overall survival in patient cohorts. *MC* mast cells, *NK cells* natural killer cells, *PC* plasma cells, *EC* eosinophilic cells; **A**–**D** patients with high MC infiltrates had a significantly prolonged overall survival (OS) in the total cohort (*n* = 812) as well as in the treatment-naive subgroup (*n* = 245); **E**–**H** this was also observable in NK cell-positive tumors in the total cohort (*n* = 818) as well as the patient cohort treated with primary surgery (total *n* = 247); **I**–**L** for PC, no significant differences in OS depending on the extent of the infiltrate were observed. **M**–**P** Patients who received primary surgery (total *n* = 249) with EC-positive tumors had a significantly prolonged OS compared to EC-negative tumors; a log-rank test was performed for statistical comparison; in case of crossing Kaplan–Meier curves, a Breslow test was used (**E**, **F**, **J**, **M**, **N**, **P**). A *p* value of *p* < 0.05 was considered statistically significant

For 842 patients (88.2%), survival data were available. To consider post-surgical complications in the very invasive procedure of esophagectomy, followed by intensive care treatment, all cases with a survival period < 1 month were excluded. The median overall survival (OS) was 22.9 months (1–233 months). In the period of clinical follow-up, 475 patients died (56.4%), 367 patients survived until censored (43.6%). Patients with a high presence of intra-tumoral MC had a significantly prolonged median OS compared to tumors with absent or sparse MC infiltrate (log-rank test: *p* < 0.001) (Fig. 2A). A prolonged survival was also observed for NK cell-positive tumors compared to NK cell-negative tumors (Breslow test: *p* = 0.004) (Fig. 2E). When sub-divided by treatment, the survival advantage was primarily observed in patients who did not receive neoadjuvant treatment: Patients with extensive MC infiltrate and NK cell-positive tumors lived longer (MC: *p* < 0.001; NK cells: *p* < 0.001) (Fig. 2B, F). Additionally, patients with EC-positive tumors and no neoadjuvant therapy had a prolonged OS (*p* = 0.009). In univariate analysis, MC and NK cells were favorable prognostic factors (MC: HR 0.57, 95% CI 0.43–0.73, *p* < 0.001; NK cells: HR 0.76, 95% CI 0.63–0.91, *p* = 0.004); No significant difference in OS was observed for plasma cells, neither for the total patient cohort nor for the patients with primary surgery or neoadjuvant treatment (Fig. 2I–L).

Patients’ age, pT, pN, AJCC grade, and the application of neoadjuvant therapy were significantly correlated to survival in univariate analyses (data not shown) and included as significant prognostic covariates into multivariate analysis: Only NK cells remained a significant favorable prognostic factor in the total cohort (HR 0.61, 95% CI 0.42–0.87, *p* = 0.007; Table 4). In the patient cohort treated with primary surgery only, NK cells remained an independent prognostic factor as well (HR 0.65, 95% CI 0.42–0.99, *p* = 0.04).

**Table 4:** Multivariate analysis for immune cell infiltrate in esophageal adenocarcinoma

MC	Total cohort			Primary surgery cohort		
	*n* events (%)	HR (95% CI)	*p* value	*n* events (%)	HR (95% CI)	*p* value
Negative/low	390 (60.0)	0.92 (0.58–1.45)	0.71	93 (53.8)	0.86 (0.52–1.422)	0.56
High	68 (41.61)			23 (33.3)		
NK cells						
Negative	286 (60.5)	0.61 (0.42–87)	**0.007**	86 (57.0)	0.65 (0.42–0.99)	**0.04**
Positive	174 (50.6)			31 (33.33)		
PC						
Negative/low	404 (57.5)	1.23 (0.76–2.12)	0.37	100 (49.0)	1.22 (0.71–2.11)	0.48
High	54 (48.6)			17 (42.5)		
EC						
Negative	390 (57.9)	0.63 (0.34–1.17)	0.14	109 (51.4)	0.77 (0.38–1.57)	0.47
Positive	65 (49.2)			9 (27.3)		

*MC* mast cells, *NK cells* natural killer cells, *PC* plasma cells, *EC* eosinophilic cells

Multivariate Cox PH analysis included, besides immune cell infiltrate, the patient’s age, tumor stage (pT), lymph node stage (pN), and AJCC grade and, in the total cohort, application of neoadjuvant treatment; a *p* value < 0.05 was considered to be significant. In patients treated with CROSS or FLOT prior to surgery, no significant prognostic impact of the immune cell extent was observed (data not shown), as already indicated by Kaplan–Meier survival curves and log-rank tests (see above Fig. 2)

### High immune cell infiltrate is correlated to longer survival in both sexes

**Fig. 3:**
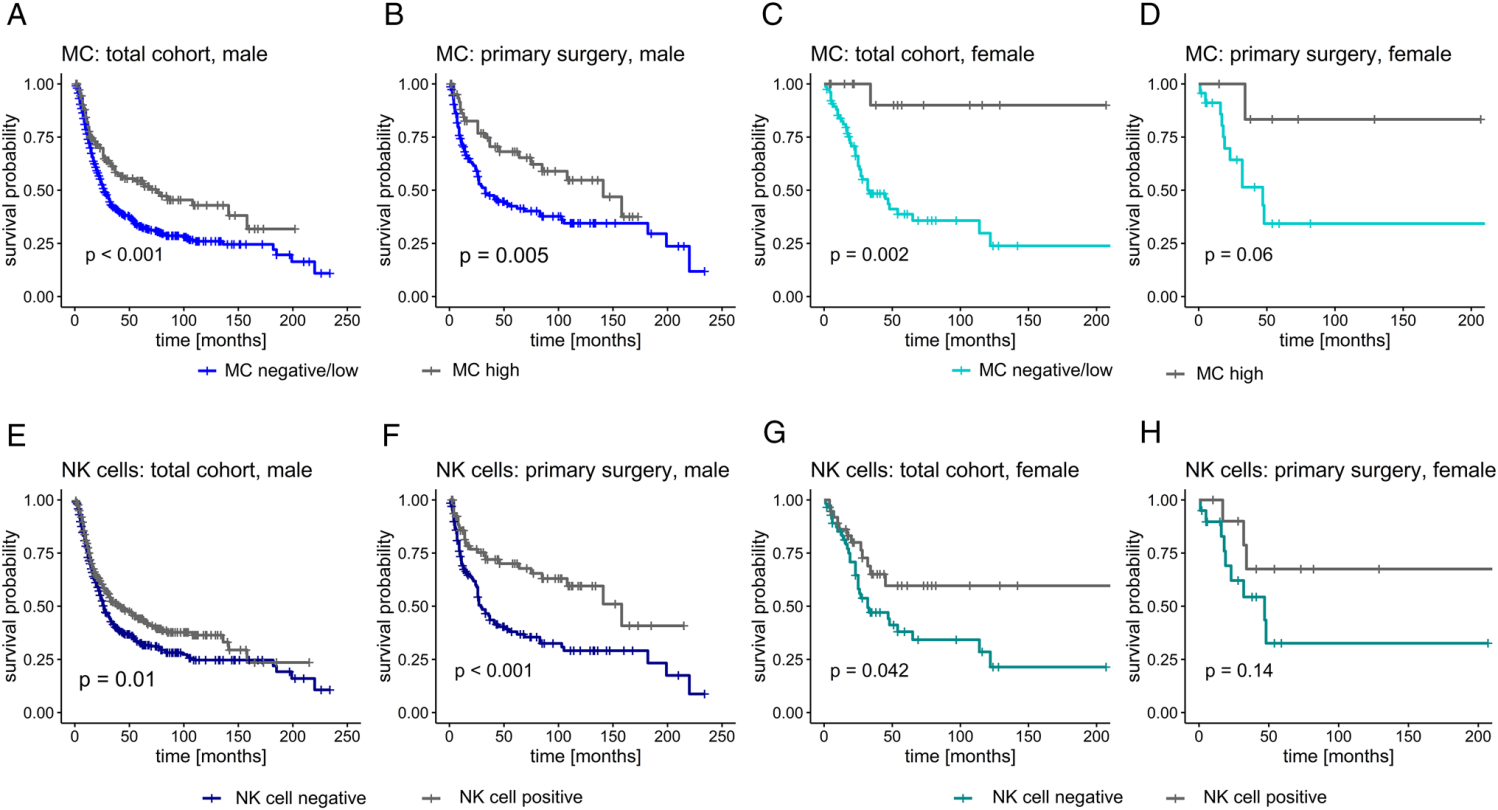
Mast cell and natural killer cell infiltrate and survival in male and female patients. **A**–**D** High amounts of mast cells (MC) were significantly associated with a prolonged overall survival in male and female total cohorts (male: *n* = 809, female: *n* = 112). This effect was also observed separately for the treatment-naïve patients (male: *n* = 269, female: *n* = 41), although only a statistical trend was observed for female patients, **D**; **E**, **F** male patients with high levels of natural killer cells (NK cells) lived significantly longer than patients with low NK cell levels. This was observed in the total cohort (*n* = 813) and the group without neoadjuvant treatment (*n* = 270); **G**, **H** in the female cohort, a significantly prolonged survival was observed in the total cohort (*n* = 113); a log-rank test was performed for statistical comparison; in case of crossing Kaplan–Meier curves, a Breslow test was used. A *p* value < 0.05 was considered statistically significant, a *p* value < 0.1 a statistical trend

When dichotomized by patients’ sex, a prolonged OS was observed in the total male as well as in the total female cohort and also in male and female patients who did not receive neoadjuvant (radio-)chemotherapy (Fig. 3). Significant differences in OS were observed in the total cohorts for MC infiltrate (male: *p* < 0.001, female: *p* = 0.002; log-rank test) and in the male cohort treated with primary surgery (*p* = 0.007; Breslow test). Male and female patients with presence of NK cells also had a significantly longer OS (Fig. 3E–H). Only in the male subgroup, the presence of NK cells remained as an independent prognostic factor (HR 0.65, 95% CI 0.44–0.96, *p* = 0.03).

### MC are a favorable prognostic factor in patients with lymph node metastasis

Lymph node metastasis and distant metastasis are of utmost important prognostic relevance in patients with esophageal cancer and were, as demonstrated above, associated with low levels or absence of MC and NK cells. Thus, we next dichotomized the patient cohort according to the lymph node status (pN0 vs. pN1, pN2, pN3) and selected a subgroup of patients with unknown M status or negative M status (Mx/M0).

**Fig. 4:**
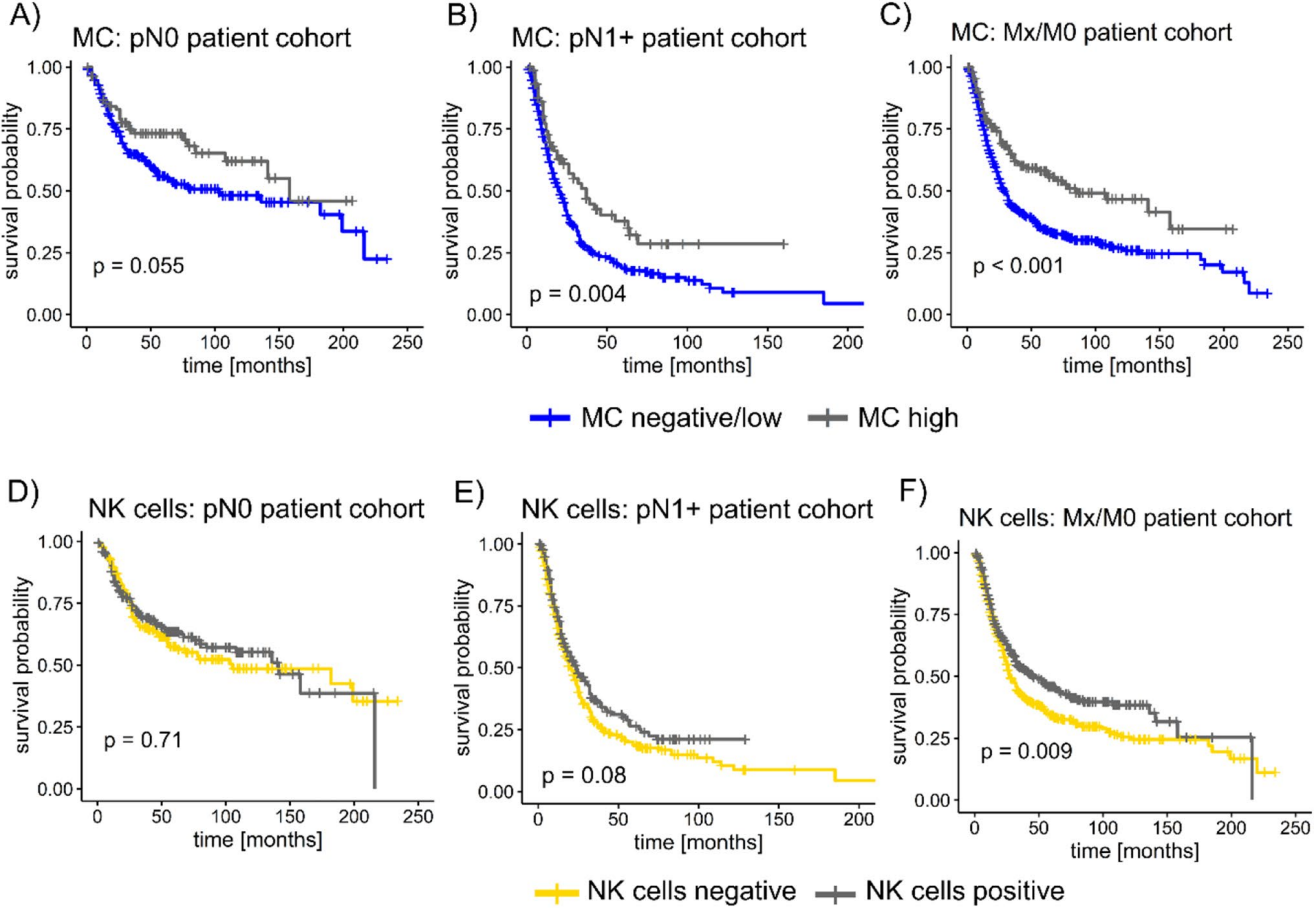
Survival analysis for subgroups with or without metastasis. The patients were dichotomized into three subgroups: A patient cohort with no lymph node metastasis (N0), with confirmed lymph node metastasis (pN+) and with unknown distant metastasis status or no distant metastasis (Mx/M0), which is usually a requirement for surgery in curative intent. **A**, **B** For both the total cohort as well as treatment-naïve tumors with and without lymph node metastasis, a prolonged overall survival (OS) was observed for mast cells (MC); this was also observed for the Mx/M0 cohort (**C**) and was significant in the pN + and Mx/M0 subgroup; **D**–**F** for natural killer cells (NK cells), the presence of NK cells was significantly correlated with a prolonged OS in the Mx/M0 cohort; a log-rank test was used with a *p* value < 0.05 considered statistically significant

High levels of MC infiltrate were associated with a prolonged OS in lymph node-negative subgroup (statistical trend, log-rank test *p* = 0.055) and lymph node metastasis (log-rank test *p* = 0.004). This was also observed in patients with Mx/M0 status (*p* < 0.001). Patients with NK cell-positive tumors and lymph node metastasis lived longer than patients with negative tumors (statistical trend, *p* = 0.08, Fig. 4E); In patients with Mx/M0 status, this difference in survival was significant (Breslow test *p *= 0.009).

In multivariate analysis, tumor stage (pT), AJCC grade, age, and the application of neoadjuvant treatment were included. A high infiltrate of MC remained an independent prognostic factor in lymph node-positive patients (Table 5). In patients with unknown distant metastatic status or M0 status, the presence of NK cells, like in the total cohort, remained an independent favorable prognostic factor (Table 5).

**Table 5:** Multivariate analysis MC and NK cell infiltrate separated by pN and M status

	*n* events (%)	HR (95% CI)	*p*
(y)pN0			
MC negative/low	106 (42.1)	0.90 (0.56–1.44)	0.66
MC high	27 (31.8)		
NK cell negative	69 (41.3)	1.0 (0.71–1.40)	0.99
NK cell positive	64 (38.6)		
(y)pN +			
MC negative/low	284 (70.5)	0.64 (0.46–0.90)	**0.009**
MC high	40 (52.6)		
NK cell negative		0.83 (0.66–1.04)	0.11
NK cell positive			
Mx/M0			
MC negative/low	372 (59.4)	1.05 (0.66–1.68)	0.84
MC high	64 (41.6)		
NK cell negative	269 (60.0)	0.62 (0.43–0.91)	**0.014**
NK cell positive	170 (50.3)		

*MC* mast cells, *NK cells* natural killer cells; multivariate Cox PH analysis included patient’s age, tumor stage (pT), AJCC grade, and application of neoadjuvant treatment; A *p* value < 0.05 was considered to be significant

## Discussion

In this study, the amounts of intra-tumoral MC, NK cells, PC, and EC in over 900 carcinoma samples were assessed, which is, to our knowledge, the largest cohort assembled for histopathological and immunohistochemical analyses of EAC yet. With a male-to-female ratio of around 6–7:1 and a median age of 63 years (27–91), our cohort accurately represents the common sex and age distribution in Caucasian European and Northern American patient cohorts (Wheeler and Reed 2012; Xie and Lagergren 2016, Strauss et al. 2020).

We demonstrated a significant association of absent or low levels of immune cells with higher tumor and lymph node stage. Tan et al. described a significant correlation of high intra-tumoral mast cells and less tumor invasion, less lymph node metastasis and an improved OS in colorectal cancer, which is exactly concurring with our findings in esophageal adenocarcinoma (Tan et al. 2005). Additionally, a favorable role of intra-tumoral mast cells has been reported for non-small cell lung cancer (Carlini et al. 2010), melanoma (Siiskonen et al. 2015), and breast cancer (Rajput et al. 2008). Interestingly, in several other studies, high MC infiltrate is associated with a poor prognosis, for example in gastric adenocarcinoma (Sammarco et al. 2019) or esophageal squamous cell carcinoma (Fakhrjou et al. 2014). The miscellaneous role of MC is commonly explained with their various functions, including unfavorable factors like angiogenesis (Norrby 2002; Marichal et al. 2013), suppression of intra-tumoral T cell response (Dalton and Noelle 2012) as well as favorable factors like innate immune cell activation (Galli et al. 2005) and direct tumor growth inhibition via mediators (Lázár-Molnár et al. 2002; Samoszuk et al. 2005).

One crucial part of the innate immune system are natural killer cells, which serve as one of the first defense lines against early-stage tumor cells and are highly involved in the tumor microenvironment regulation (Wu et al. 2020). Al-Shibli et al. demonstrated natural killer cells to be a favorable prognostic factor in non-small cell lung cancer (Al-Shibli et al. 2009). Coca et al. reported a correlation between extensive NK cell infiltrate and prolonged overall survival and disease-free survival in colorectal adenocarcinoma, respectively. Additionally, NK cell infiltrate was an independent prognostic factor in advanced stage (Coca et al. 1997). In this study, a prolonged survival for NK cell-positive tumors in the total cohort and the primary resection cohort was observed—furthermore, these tumors were less infiltrative, less frequently spread to lymph vessels, lymph nodes, and distant sites of the body. These findings are in perfect accordance with the results of Svensson et al. who demonstrated that NK cells are an independent prognostic factor in esophageal adenocarcinoma and are associated with prolonged overall survival (Svensson et al. 2017). We could observe and confirm the same prognostic favorable impact in a much larger cohort and under consideration of other pre-treatments (CROSS, FLOT), demonstrating that the prognostic impact is carried by the treatment-naïve and total cohort.

We noticed that high levels of plasma cells were significantly correlated to less extensive tumor stage and lymph node metastasis occurrence. This was described as well by Fristedt et al. who demonstrated that only a subset of immunoglobulin kappa chain-expressing plasma cells had a significant prognostic impact on overall survival and disease-free survival, not a general plasma cell infiltrate (Fristedt et al. 2016). We did not further analyze plasma cell subgroups; however, we could confirm in our cohorts as well that the total population of plasma cells is not significantly correlated to longer survival. Another study included 210 cases of adenocarcinoma of the gastroesophageal junction and described a significant prognostic trend for plasma cells and a clear significant prognostic impact for general B cell infiltrate (Knief et al. 2016). This study, however, did not include the lymph node status in the multivariate analysis, which was the strongest prognostic factor in nearly all of our multivariate analyses and is considered to be a crucial, if not the most important prognostic factor for overall survival in patients with EAC (Kayani et al. 2011, Stiles et al. 2011).

While several studies have shown a favorable prognostic significance of tumor-associated eosinophils in various solid tumors, including ESCC and colorectal cancer (Prizment et al. 2016; Hu et al. 2020), such consideration is lacking for EAC: Here, we demonstrated a significant association of EC positivity and less infiltrative disease as well as less frequent lymph node involvement. To our knowledge, this is the first and very extensive study describing a significant correlation between tumor aggressiveness and intra-tumoral eosinophils in esophageal adenocarcinoma.

Since lymph node involvement was the strongest prognostic factor for reduced OS in the multivariate analyses, we sub-divided the patient cohort in subgroups with and without lymph node metastasis, respectively. Interestingly, intra-tumoral MC were significantly associated with prolonged OS and were a favorable prognostic factor in patients with lymph node metastasis. The importance of lymph node status is highlighted by the recent international European guidelines for esophageal cancer: If a lymph node metastasis is diagnosed in staging, the patients receive neoadjuvant treatment and, if vital cancer cells are observed after surgery, nivolumab (Kelly et al. 2021; Obermannova et al. 2022). However, as encouraging as it might seem in a deadly cancer like EAC, when residual vital tumor in lymph nodes was diagnosed after surgery, nivolumab reached a disease-free survival of 19.4 vs. 11.1 months in the placebo group (Kelly et al. 2021), which demonstrates the urgent need for additional therapeutic approaches. Interestingly, EAC patients with lymph node metastasis after neoadjuvant treatment and surgery display a worse prognosis compared to ESCC (Depypere et al. 2018). This once more highlights the different biological and clinical behaviors of these distinct tumor entities, which remain poorly understood. We could furthermore demonstrate that the presence of NK cells was a favorable prognostic factor in patients with unknown M status or confirmed absence of distant metastasis. While this sub-division and detailed analysis might seem contra-intuitive at first, the absence of distant metastasis is one, if not the most important deciding factor whether a patient can be treated with curative intent according to German guidelines (Monig et al. 2018). Patients with absence of NK cells significantly more often had distant metastasis, lymph vessel invasion, and lymph node metastasis and might, therefore, profit from a closer follow-up, aiming for early detection of distant metastasis.

While this study is retrospective, it still comprises a very large number of patients; nevertheless, usual biases of retrospective study designs are not excluded even in this case. The majority of the analyses was performed on tissue micro arrays (TMAs): The tissue core diameter of 1.2 mm was chosen in such a way that the average number of carcinoma cells assessed roughly corresponds to an amount of tumor cells that can also be expected in endoscopic biopsies. Due to this design, an assessment of the adjacent extra-tumoral inflammatory cells was not possible. However, we performed exemplary large-area sections to validate our results and are convinced that the chosen TMA technique provides sufficiently representative results. It will be interesting in the future to correlate the significance of inflammatory cell composition (especially mast cells) on endoscopically obtained biopsies with prognosis (and lymph node metastasis probability).

## Conclusion

In this study, we comprehensively demonstrated the correlation of intra-tumoral immune cell infiltrate and a less aggressive and infiltrative disease. Most of the biological mechanisms leading to these observations, however, involve a complex spectrum of immune cell interactions and, especially for esophageal adenocarcinoma, are not fully elucidated yet. However, the immune cell infiltrate in esophageal cancer is a promising key to a better understanding of the tumor and its biology, the prognostic, and also the potential therapeutic implications.

## Data Availability

The data that support the findings of this study are available from the corresponding author, AGS, as well as AQ, upon reasonable request.
